# Supplementary material for: Postpartum hemorrhage risk is driven by changes in blood composition through pregnancy
Source: Sci Rep. 2021 Sep 28;11:19238. doi: 10.1038/s41598-021-98411-z (PMC8478943; doi:10.1038/s41598-021-98411-z)
Supplement: Supplementary file 1 — Supplementary Figure S1. [file 41598_2021_98411_MOESM1_ESM.pdf]

Supplementary Information

Postpartum hemorrhage risk is driven by changes in blood composition through pregnancy

Matthew R. Robinson, Marion Patxot, Miloš Stojanov, Sabine Blum, David Baud

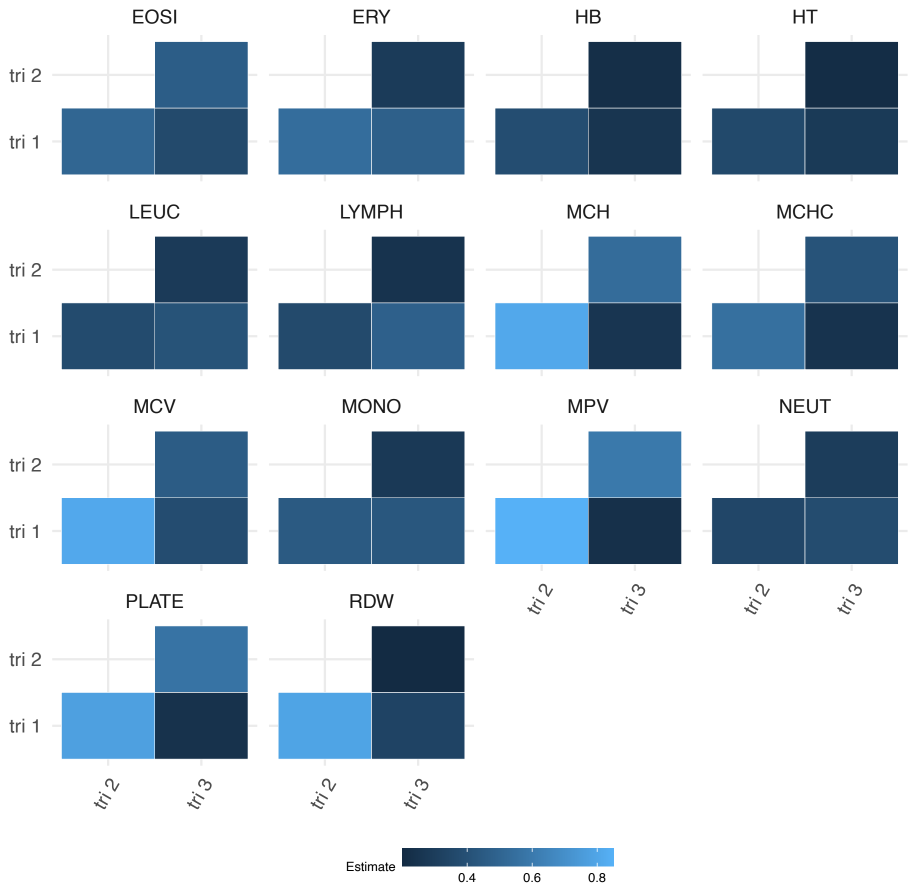

Figure S1. Path coefficients of trimester-specific complete blood count values. Path relationships between complete blood count measures indicating the strength of relationship (on the correlation scale) of measures recorded across the three trimesters of pregnancy. Measure abbreviations: absolute eosinophil (EOSI), lymphocyte (LYMPH), monophil (MONO) and neutrolphil (NEUT) levels; leukocyte count (LEUC), erythrocyte count (ERY), hematocrit (HT), hemoglobin level (HB), mean corpuscular hemoglobin (MCH) and the concentration (MCHC), mean corpuscular volume (MCV), mean platelet volume (MPV), platelet count (PLATE), and red cell distribution width (RDW)
